# Prevalence and type-specific distribution of human papillomavirus infection among women in mid-western rural, Nepal- A population-based study

**DOI:** 10.1186/s12879-018-3175-9

**Published:** 2018-07-20

**Authors:** Niresh Thapa, Muna Maharjan, Girishma Shrestha, Narayani Maharjan, Marcia A. Petrini, Na Zuo, Can He, Jing Yang, Mengfei Xu, Caiyun Ge, Ziye Song, Hongbing Cai

**Affiliations:** 1 Department of Gynecological Oncology, Zhongnan Hospital of Wuhan University, Hubei Cancer Clinical Study Center, Hubei Key laboratory of Tumor Biological Behaviors, Wuhan, 430071 People’s Republic of China; 2 Karnali Academy of Health Sciences, Jumla, Nepal; 3 Zhongnan Hospital of Wuhan University, HOPE School of Nursing, Wuhan, Hubei China; 4 Patan Hospital, Patan Academy of Health Sciences, Patan, Nepal; 5 Department of Clinical Laboratory Science, Zhongnan Hospital of Wuhan University, Wuhan, Hubei China; 6 Faculty of Nursing, Chiang Mai University, Chiang Mai, Thailand

**Keywords:** Cervical cancer, Genotype, HPV infection, Nepal, Prevalence

## Abstract

**Background:**

Cervical cancer is the most common cancer among women in Nepal. The prevalence of human papillomavirus (HPV) 16 and or HPV 18 among women with cervical pre-cancer and cancer is higher than the incidence of HPV in the world population. The population-based epidemiological data of HPV in the general population in most parts of the country remains unknown. The objective of this study was to assess the prevalence and type distribution of HPV infection and association of abnormal cytology with high risk HPV infection among women in mid-western rural, Nepal.

**Methods:**

A population-based cross sectional study was conducted in Jumla, one of the most remote districts in Nepal. A total of 1050 cervical samples were collected from married and non- pregnant women aged 20–65 years during mobile Cervical Cancer Screening Clinics conducted from May 2016 to January 2017. The presence of HPV DNA was firstly confirmed by HPV consensus PCR using PGMY09/PGMY11 designed primers, then HPV positive samples were further genotyped by the membrane hybridization method to detect the 21 high-risk HPV (HR-HPV) and low-risk HPV types. The prevalence of HR-HPV among women with normal and abnormal cytology was calculated. Data were analyzed using SPSS software for Windows. *P* < 0.05 was considered statistically significant.

**Results:**

A total of 998 women were eligible for this study with the mean age 32.6 ± 8.6 years, and the mean marital age was 16.7 ± 3.8 years. The overall prevalence of HPV infections was 19.7%. HR-HPV and low-risk HPV were 11.7 and 8.7% respectively. The six most common HR-HPV types were HPV16, 39, 58, 33, 51 and 18. HR-HPV infection among the women with abnormal and normal cytology was of 27.3 and 10.8% respectively.

**Conclusions:**

There was a higher prevalence of HR-HPV infection among women living in Jumla than other parts of Nepal. This study provides preliminary information on overall HPV and type-specific HR-HPV prevalence, HR-HPV 16, 39, 58, 33, 51, and 18 are the most prevalent genotypes in this region. The data contribute to the epidemiological knowledge about HPV and type-specific HR-HPV genotypes prevalence in mid-Western Nepal.

## Background

Worldwide, the prevalence of human papillomavirus (HPV) 16 and or HPV 18 among women with low grade squamous Intraepithelial Lesions (LSIL), high-grade squamous intraepithelial lesions (HSIL) and cervical cancer is 25.8, 51.9 and 69.4% respectively [1]. The burden of HPV infection is significantly high about 10 million new cases of HSIL, and 30 million new cases of LSIL are diagnosed every year. The potential risk of HPV for cervical cancer is higher than Hepatitis B virus (HBV) is for liver cancer and even smoking is for lung cancer [2]. HPV infection is regarded as the well-established cause of cervical cancer now and a risk factor for other anogenital as well as head and neck cancers [1].

Cervical cancer is the most common cancer among women in Nepal. Cervical cancer demonstrates a decreasing trend in more developed countries, but consistently remains the number one female cancer of incidence and mortality in Nepal. According to the Information Center on HPV and Cancer (ICO), Nepal reports a prevalence of HPV 16 and or HPV 18 among women with LSIL, HSIL, and cervical cancer are 30.2, 63.4, and 80.3% respectively. The prevalence of HPV infection in Nepal is higher than in the world population [3–5].

Human genital HPV types are categorized according to the epidemiologic association and potential risk of cervical cancer. The eight most common types high risk (HR) HPV (HPV-16, 18, 31, 33, 35, 45, 52, 58) and relatively less common four types (HPV-39, 51, 56, 59) found in cervical cancer were classified in group 1. Probably carcinogenic to human’s HPV-68 was classified in group 2A. The remaining types of HPV 26, 53, 66, 67, 70, 73, 82 of the high-risk alpha species were classified in group 2B as “possibly carcinogenic” [6, 7].

The actual distribution of HPV is not known for some resource-limited countries, including Nepal [8]. Studies reporting the epidemiologic information on HPV in Nepal are sparse. Sherpa et al. in 2010 indicated that among the general population the overall prevalence of HPV was 8.6% including 6.1% for high-risk HPV [9]. Among the women with invasive cervical cancer, the incidence of HPV 16/18 infection was around 91% [9] and 70% [10]. Similarly, another study conducted by Shakya et al. [11] in the rural site of Kavre district found that the overall HPV prevalence was 14.4% including 7.9% of HR-HPV, whereas 9.6% of HR-HPV infection was reported in a study from far-western region of Nepal [12]. Furthermore, a study conducted by Bhatta et al. in 2017 among Nepali and Bhutanese women living in eastern Nepal reported the prevalence of 8.9% HR-HPV infection [13]. In the presence of co-factors, persistent infection with an oncogenic HPV is necessary to develop cervical cancer [14]. Therefore, the HPV test has emerged as an essential screening tool. HPV infection status is thus significant in cervical cancer screening and prevention strategy [15, 16].

Reproductive health indicators in Jumla are below the level than general Nepali women. The prevalence of female adolescent marriage is almost 83%; the total fertility rate is 4.2, literacy among women is 41% in Jumla [17, 18]. The population based epidemiological data of HPV in the general population in most parts of the country is still unknown [11]. So far, we know this was the first population-based study conducted in such remote area, Jumla. The objective of this study was to assess the prevalence and type-specific distribution of HPV infection and association of abnormal cytology with high-risk HPV infection among apparently healthy women in mid-western rural, Nepal. This study intends to give a sound basis for future strategic plans for the prevention of cervical cancer.

## Methods

### Study setting

A population based cross sectional study conducted in Jumla. According to National Population and Housing census 2011, the total population of Jumla is 108921, and the overall, married women registration is 27309. Jumla is one of the most remote districts in Nepal with women who have a high illiteracy rate, high poverty, low economic development, and inadequate transportation facility [17, 18].

### Sample collection

Ethical approval was obtained from the Nepal Health Research Council (NHRC), Nepal and Department of Gynecological Oncology, Second Clinical College of Wuhan University, China. The study participants were recruited from the different mobile cervical cancer screening programs conducted in Jumla, in 2016 and 2017. Inclusion criteria in this study were: aged 20–65 years married, non-pregnant, apparently healthy, asymptomatic women, with no history of cervical cancer. The exclusion criteria were women who were menstruating or did not want to participate in the study. Eligible women were counseled about the research and informed that they could withdraw from the study at any time with no impact on the routine care. A written informed consent was obtained from eligible participants with either a signature or thumbprint. Cervical cells of 1050 women were collected using cytobrush (Hybribio® Limited, China) from the eligible women who were in follow up for our previous screening test (cytology) report.

Cytology was done 4 weeks earlier and reported according to the Bethesda system [19]. For the analysis purpose, the cytology report was categorized into normal and abnormal. Routine cervical cytology included negative for intraepithelial lesion or malignancy (NILM) and inflammatory changes. Abnormal cervical cytology included atypical squamous cells of undetermined significance (ASC-US), low-grade squamous intraepithelial lesion (LSIL), high-grade squamous intraepithelial lesion (HSIL) and squamous cell carcinoma (SCC).

### DNA extraction PCR and HPV genotyping

DNA extracted in Intrepid Cancer Diagnostics, Kathmandu using the QIAamp DNA Mini kit (QIAGEN Germany) according to the manufacturer’s instructions. Extracted DNA samples were stored at − 20 °C until transported on dry ice to the Zhongnan Hospital of Wuhan University, Hubei Cancer Clinical Study Center, Hubei Key Laboratory of Tumor Biological Behaviors, Wuhan, China for HPV DNA analyses.

The presence of HPV DNA was firstly confirmed by HPV consensus PCR using PGMY09/PGMY11 designed primers to amplify a fragment of the HPV L1 gene visible at 450 bp and Human Leukocyte Antigen (HLA) band at 230 bp as described in ‘Human papillomavirus laboratory manual’ published by World Health Organization [20]. Detection of HLA confirmed that DNA had been extracted in sufficient amount. Amplicons were detected after the agarose gel electrophoresis and Ethidium bromide staining under the UV transillumination. Gel electrophoresis analysis was done to limit the only HPV positive samples for further genotyping by membrane hybridization (21 HPV GenoArray Diagnostic Kit, Hybrobio® Limited, China) to detect the following 21 HPV types: HPV6, 11, 16, 18, 31, 33, 35, 39, 42, 43, 44, 45, 51, 52, 53, 56, 58, 59, 66, 68, 81 (equivalent to CP8304) according to the manufacturer’s instruction. High risk HPV (HR-HPV) types included in this study were HPV16, 18, 31, 33, 35, 39, 45, 51, 52, 53, 56, 58, 59, 66, and 68 [7]. All other HPV types were considered low-risk HPV (LR-HPV).

### Statistical analysis

Data were analyzed using SPSS software for Windows (version 16.0). The distribution of characteristics of participants was presented as numbers, percentage and mean. Odds ratios (ORs) and their 95% confidence intervals (CIs) were obtained using univariate analysis to assess the effect of characteristics in HPV infection. *P* < 0.05 was considered statistically significant.

## Results

Altogether 1050 samples were collected; 20 samples were not adequate for DNA extraction, and 32 samples had no HLA band visible under UV transillumination after Gel Electrophoresis of PCR product suggesting invalid for further analysis. Table 1 shows the distribution of HR-HPV according to the socio-demographic and reproductive health characteristics. Among 998 eligible women, the mean age was 32.6 ± 8.6 years; the mean marital age was 16.7 ± 3.8 years. There were around 40% of women married before the age of 15 years. About 50% of women had more than three children. Forty-one percent of women were illiterate, and among the literate group more than half just had an informal education which means they can hardly read or write. Almost 16% of women were a current smoker. A significant number of women (14.3%) reported that they had sexually transmitted infection (STI) in the past and about 12% of the participant’s husband had multiple marriages or multiple sexual partners. There were 5 cases of HIV positive women. HR-HPV was most prevalent in the younger age group from 20 to 29 years, and in the same way more common on those who marry before the age of 20 years. The HR-HPV Positive rate was increased correspondingly with the number of pregnancies and number of childbirths.

**Table 1:** Characteristics and distribution of high-risk Human Papillomavirus (HR-HPV) infection among women living in mid-western rural Nepal *N* = 998

Characteristics	Number (%)	HR-HPV (f/n)	HR-HPV (%)
Age			
20–29	403 (40.4)	58/403	14.4
30–39	351 (35.2)	23/351	6.5
40–49	192 (19.2)	26/192	13.5
50–59	42 (4.2)	5/42	11.9
Above 60	10 (1.0)	3/10	30
Mean ± SD	32.6 ± 8.6		
Marital age			
≤ 15	393 (39.4)	41/393	10.4
16–20	513 (51.4)	62/513	12.1
21–25	61 (6.1)	5/61	8.2
≥ 26	31 (3.1)	7/31	22.6
Mean ± SD	16.7 ± 3.8		
Number of pregnancy			
0	35 (3.5)	0/35	0
1–2	278 (27.9)	30/278	10.8
3–4	425 (42.5)	44/425	10.3
≥ 5	260 (26.1)	41/260	15.7
Number of children			
0	42 (4.2)	3/42	7.1
1–2	470 (47.1)	39/470	8.3
3–4	411 (41.2)	54/411	13.1
≥ 5	75 (7.5)	19/75	25.3
Education			
Illiterate	410 (41.1)	49/410	11.9
Literate	588 (58.9)	66/588	11.2
Education level (*n* = 588)			
Informal education	295 (50.2)	24/295	8.1
Primary level	98 (16.7)	7/98	7.1
Secondary level	101 (17.2)	21/101	20.8
Higher secondary level and above	94 (15.9)	14/94	14.9
Smoking			
Smoker	158 (15.8)	14/158	8.8
Non-smoker	840 (84.2)	101/840	12.0
Participant’s multiple marriage/ sexual partners			
Present	70 (7.0)	5/70	7.1
Absent	928 (93.0)	110/928	11.8
Husband’s multiple marriage / sexual partners			
Present	118 (11.8)	23/118	19.5
Absent	880 (88.2)	92/880	10.4
Oral contraceptives use			
Users	95 (9.5)	5/95	5.3
Non-users	903 (90.5)	110/903	12.2
Co-morbidity (*n* = 148)			
Sexually transmitted infection	143 (14.3)	19/143	13.3
Human immunodeficiency virus	5 (0.5)	3/5	60

There were 197 (19.7%) HPV positive cases detected from 998 acceptable samples. High and low risk HPV were 115 (11.5%) and 82 (8.2%) respectively. In total, 13.6% women had single-type infections, and 6.1% had multiple-type infections. HPV 16 and HPV 39 were the most common multiple infections. The six most common HR-HPV types were HPV16 (6.7%), 39 (4.8%), 58 (2.8%), 33 (2.6%), 51 (1.4%) and 18 (1.2%). HPV 6 (0.6%) was the most common LR-HPV. There was no positive case of HR-HPV 59. The number of other uncharacterized LR-HPV type was 72 (Table 2).

**Table 2:** Type-specific distribution of Human Papillomavirus (HPV) infection among women living in mid-western rural Nepal *N* = 998

Types	HPV infection		Total	Percentage
	Single	Multiple		
Overall HPV Positive	136	61	197	19.7
High-Risk HPV^a^				
16	19	48	67	6.7
18	5	7	12	1.2
31	1	5	6	0.6
33	7	19	26	2.6
35	3	0	3	0.3
39	11	37	48	4.8
45	1	0	1	0.1
51	5	9	14	1.4
52	1	3	4	0.4
53	3	5	8	0.8
56	3	5	8	0.8
58	6	22	28	2.8
59	0	0	0	0
66	0	3	3	0.3
68	3	0	3	0.3
Low-risk HPV^a^				
6	5	1	6	0.6
11	3	0	3	0.3
42	0	0	0	0
43	0	0	0	0
44	0	0	0	0
81	3	1	4	0.4
Other low-risk type	72	–	72	7.2

^a^Some women had multiple infections so the frequency of high-risk and low-risk HPV is not the sum of overall HPV positive

**Fig. 1:**
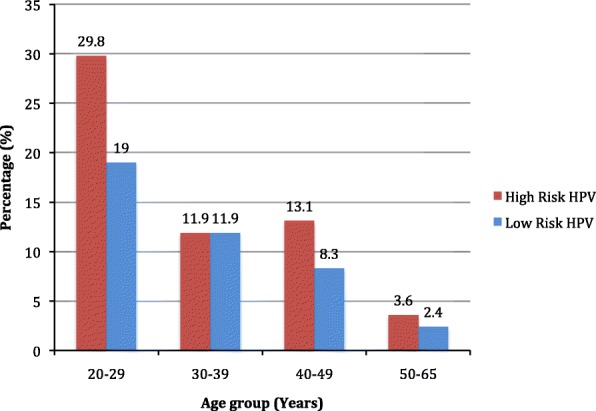
Human Papillomavirus (HPV) infection according to the age group of women living in mid-western rural Nepal

**Fig. 2:**
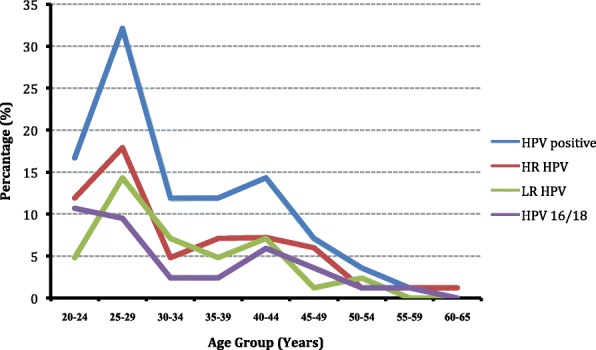
Trends of Human Papillomavirus (HPV) infection according to the age group of women living in mid-western rural Nepal

Figure 1 illustrates that HR-HPV is more prevalent in the younger age group. In the age group of 20–29 years, 29.8% of HR HPV infection occurred, whereas only 3.6% of HR HPV infection presented in 50–65 years’ age group. There were ‘two peaks’ in the prevalence pattern of the overall HPV and HPV16/18 infections; the first peak was at the age group of 25–29 years and the second small peak was at the age group of 40–44 years (Fig. 2).

Table 3 presents the association between variables and HR-HPV infection. The relationship between HR-HPV and the variables of age, marital age, number of pregnancies, number of childbirths, education status, smoking, participant’s multiple marriages or multiple sexual partners, husband’s multiple marriages or multiple sexual partners, contraceptive use, HIV status and STI status were analyzed with the use of odds ratio (OR). Chi-square test was used for the statistical significance. The HR-HPV was significantly associated with age, number of pregnancy, number of children, husband’s multiple marriages or multiple sexual partners and participant’s HIV status (*p* < 0.05).

**Table 3:** Univariate analyses of potential risk factors for high-risk Human Papillomavirus (HR-HPV) in women living in mid-western rural Nepal

Characteristics	HR-HPV		OR (95% CI)	*p*-value
	Positive	Negative		
Age				
≤ 45 years	96	803	0.50 (0.29–0.86)	0.013
≥ 46 years	19	80		
Marital age				
≤ 19 years	101	754	1.23 (0.68–2.22)	0.483
≥ 20 years	14	129		
Number of pregnancy (*n* = 529)				
≥ 4	58	392	3.74 (1.14–12.27)	0.029
0 or 1	3	76		
Number of children (*n* = 630)				
≥ 3	73	413	1.96 (1.03–3.72)	0.039
0 or 1	12	133		
Education status				
Illiterate	49	361	1.07 (0.72–1.59)	0.723
Literate	66	522		
Smoking				
Smokers	14	144	0.71 (0.39–1.27)	0.255
Non-smokers	101	739		
Participant’s multiple marriage/ sexual partners				
Present	5	65	0.57 (0.22–1.45)	0.239
Absent	110	818		
Husband’s multiple marriage/sexual partners				
Present	23	95	2.07 (1.25–3.43)	0.004
Absent	92	788		
Oral contraceptive use				
Users	5	90	0.40 (0.15–1.00)	0.051
Non-users	110	793		
HIV				
Present	3	2	11.82 (1.95–71.54)	0.007
Absent	112	883		
STI				
Present	19	124	1.21 (0.71–2.05)	0.476
Absent	96	759		

Abnormal cytology reports were 44 in 998 women. HR-HPV infection among women with abnormal and normal cytology was 27.3 and 10.8% respectively. Positive HR-HPV was significantly higher in women with abnormal cytology. The abnormal cytology for women with LSIL, ASC-US, HSIL, and SCC had HR-HPV infection rates of 25, 13.3, 40 and 75% respectively (Table 4).

**Table 4:** Prevalence of high-risk Human Papillomavirus (HR-HPV) infection among women with normal and abnormal cervical cytology

Cytology result	HR-HPV		OR (95% CI)	*p*-value
	Positive	Negative		
Abnormal (*n* = 44)	12 (27.3)	32 (72.7)	3.09 (1.54–6.20)	0.001
Normal (*n* = 954)	103 (10.8)	851 (89.2)		
Total cytology (*n* = 998)	115 (11.5)	883 (88.5)		
Abnormal cytology (*n* = 44)				
LSIL (*n* = 20)	5 (25.0)	15 (75.0)		
ASC-US (*n* = 15)	2 (13.3)	13 (86.7)		
HSIL (*n* = 5)	2 (40.0)	3 (60.0)		
SCC (*n* = 4)	3 (75.0)	1 (25.0)		

## Discussion

A well-established fact is that HR-HPV has a vital role in cervical cancer, which induces transformation of cervical epithelial cells into precancerous lesions and slowly into cancer [21]. About 84% of the cervical cancer burden is in the developing countries and especially in low resource areas. Though cervical cancer is the most common female malignancy in Nepal, a lack of proper prevention strategies exists to curb cervical cancer through screening, early treatment or vaccination. No effective screening programs have been established. However, some progress has been observed recently.

This study revealed prevalence rates of HPV infections (19.7%) and HR-HPV (11.7%). The HPV infection rate was higher in comparison to other previous studies done in Nepal which ranged from 8.6 to 14.4% [9, 11–13]. The findings of this study represent higher than the worldwide HPV prevalence of 10.4% and the corresponding estimate of 8.0% in Asia [22]. The higher incidence of HPV in Jumla may be attributed to the following risk factors: higher proportion of younger participants (age of 20–29 years participants were 40.4% of the sample), early marriage (marital age of 91% of the women was ≤20), multiple numbers of pregnancies, multiple children, risky sexual behaviors (multiple marriages, many sexual partners, higher proportion of sexually transmitted infections). Moreover, another important reason for the higher prevalence of HPV was probably due to the detection of HPV L1 gene by PGMY 09/11 primer system, which is more sensitive than GP5+/6+ or MY09/11 and able to amplify a spectrum of more than 30 genital HPV types [23]. Most of the studies conducted in China stated the prevalence of HPV was higher and varied by region ranging from 18.42 to 31.94% while in India reported a relatively lower rate (2.4 to 14%) [3, 24].

The highest prevalence of HR-HPV infection was in women of 20–29 years of age. The rate of HR-HPV infections declined with age, which is similar to a finding with a previous study [25]. In contrast to this study, a report from the rural area of China reflected HR-HPV and HPV 16/18/45 were at the peak in the age group of 55–59 years [26]. We also observed that the proportion of HR-HPV infection was higher among the women who had multiple children (*p* = 0.039) and multiple pregnancies (*p* = 0.029). Illiterate women tend to have a slightly elevated rate of HR-HPV infection compared with women who are literate, but there was no statistical significance. Previous studies have reported that high-risk sexual behavior such as multiple sexual partners is a critical factor of HPV infection in women [27], which is similar to the present study findings. Early marital age before 19 years was weakly associated with HR-HPV infection (*p* = 0.483). Though there were few cases of HIV positive participants, there was a strong association with HR-HPV infections, which was statistically significant (*p* = 0.007). The findings are similar to a previous report [28].

In Jumla, the HPV 16 is the most common genotype followed by HPV 39. HPV 18 was in 6th position in the order. The top five HR HPV genotypes in this sample were 16, 39, 58, 33 and 51. However, a study conducted by Bhusal et al. among 44 women diagnosed with invasive cervical cancer found HPV16 was the most common HR-HPV followed by HPV18 [10]. Sherpa et al. reported the most common high-risk types among women with normal cytology were HPV16, 58, 56, 18, and 52. Meanwhile, Shakya et al. reported HPV-18, 51, 59, 31 and 16 as the five most common HPV [9, 11]. So, it is clear that there are variations in the prevalence of HPV infection frequency within Nepal. Nevertheless, HPV 16 and 18 seem to be the most common. In the South Asian, HPV 16 is the most frequently detected genotype followed by HPV 58 while HPV 18 is common in Western countries. Other common HR HPV reported in the South Asian countries are 52, 18, and 31 in different orders of frequency [29, 30]. Worldwide, the most common HPV types in women are 16, 18, 31, 58, and 52, which is about 50% of all HPV infections [22].

Two types of vaccine against HPV are licensed for use in the United States by the FDA. A commonly used quadrivalent vaccine, Gardasil® covers against HPV types 6, 11, 16, and 18. A newer vaccine, Gardasil 9, was approved by the United States Food and Drug Administration (US FDA) in 2014 and protects against HPV types 6, 11, 16, 18, 31, 33, 45, 52, and 58 [31]. A pilot vaccination program was launched among 1096 school girls in Nepal giving quadrivalent human papillomavirus (types 6, 11, 16, 18) recombinant vaccine (Gardasil; Merk & Co.). A post-vaccine study reported the vaccine was safe with high acceptability in Nepali school girls but needed longer follow up to determine the long term vaccine effect [32]. Based on our findings even Gardasil 9 would cover only around 64% of women of Jumla since it does not include HPV 39, which was the second most common genotype in the present study sample. Therefore, from the ‘cost and benefit’ point of view, vaccination is not an ideal option in low resource countries. Thus, a simple, acceptable and sustainable screening method for cervical cancer screening provides greater efficacy.

The findings of this study suggest that abnormal cytology is strongly associated with HR-HPV infection. When the severity of disease is higher from LSIL to SCC, HR-HPV the infection rate also increased from 25 to 75%. The trend and percentage of the current findings are similar to previous studies [3, 11].

As a cross-sectional study, the prevalence of HPV infection in the rural region was obtained. Further longitudinal studies are recommended to study the relationship of the persistence HPV infection and other co-factors such as early marriage (early sexual debut), multiple pregnancies, multiple numbers of children, “chaupadi” tradition (poor harmful practice during menstruation), smoking, risky sexual behaviors and low socioeconomic status with anogenital cancers. Purposive sampling technique was used to collect a sample from the participants in a cervical cancer screening camp. Thus, the sample in this study may not be a reflection of all the Nepali women. However, the results provide valuable preliminary information on overall HPV prevalence and distribution of HR-HPV Genotype in a rural population.

## Conclusions

Women living in a mid-western rural region of Nepal reflect a higher prevalence of HR-HPV infection than other areas of Nepal. This study provides preliminary information on overall HPV and type-specific HR-HPV prevalence; HR-HPV 16, 39, 58, 33, 51, and 18 are the most prevalent genotypes in this region. The data contribute to the epidemiological knowledge about HPV and type-specific HR-HPV genotypes prevalence in mid-Western Nepal.

## Abbreviations

ASC-US: Atypical squamous cells of undetermined significance
HIV: Human immunodeficiency virus
HPV: Human papillomavirus
HR: High-risk
HSIL: High-grade squamous intraepithelial lesion
LR: Low-risk
LSIL: Low-grade squamous intraepithelial lesion
SCC: Squamous cell carcinoma
STD: Sexually transmitted disease

## References

[1] Bruni L, Barrionuevo-Rosas L, Albero G, Serrano B, Mena M, Gómez D, Muñoz J, Bosch FX, de Sanjosé S. ICO/IARC Information Centre on HPV and Cancer (HPV Information Centre). Human Papillomavirus and Related Diseases in the World. Summary Report 27 July 2017. [cited 2017 Dec 17]. Available from: http://www.hpvcentre.net/statistics/reports/XWX.pdf.

[2] Franco EL, Harper DM. Vaccination against human papillomavirus infection: a new paradigm in cervical cancer control. Vaccine. 2005;23(17-18):2388–2394. Available from: http://citeseerx.ist.psu.edu/viewdoc/download?doi=10.1.1.320.4542&rep=rep1&type=pdf. 10.1016/j.vaccine.2005.01.016 15755633

[3] Bruni L, Barrionuevo-Rosas L, Albero G, Serrano B, Mena M, Gómez D, Muñoz J, Bosch FX, de Sanjosé S. ICO/IARC Information Centre on HPV and Cancer (HPV Information Centre). Human Papillomavirus and Related Diseases in Nepal. Summary Report 27 July 2017. [cited 2017 Dec 17]. Available from: http://www.hpvcentre.net/statistics/reports/NPL.pdf.

[4] Pun CB, Pradhananga KK, Siwakoti B, Subedi K, Moore MA (2016). Malignant neoplasm burden in Nepal - Data from the seven major cancer service hospitals for 2012. Asian Pacific J Cancer Prev.

[5] Ferlay J, Soerjomataram I, Ervik M, Dikshit R, Eser S, Mathers C, et al. GLOBOCAN 2012: Estimated Cancer Incidence, Mortality and Prevalence Worldwide in 2012 [Internet]. GLOBOCAN 2012 v1.0. 2013 [cited 2016 Jan 10]. Available from: http://globocan.iarc.fr/Pages/fact_sheets_cancer.aspx

[6] De Villiers EM, Fauquet C, Broker TR, Bernard HU, Zur Hausen H (2004). Classification of papillomaviruses. Virology.

[7] Bouvard V, Baan R, Straif K, Grosse Y, Secretan B, El GF (2009). A review of human carcinogens-Part B: biological agents. Lancet Oncol.

[8] De Sanjose S, Quint WGV, Alemany L, Geraets DT, Klaustermeier JE, Lloveras B (2010). Human papillomavirus genotype attribution in invasive cervical cancer: a retrospective cross-sectional worldwide study. Lancet Oncol.

[9] Sherpa ATL, Clifford GM, Vaccarella S, Shrestha S, Nygård M, Karki BS (2010). Human papillomavirus infection in women with and without cervical cancer in Nepal. Cancer Causes Control.

[10] Bhusal CL, Manandhar S, Singh M, Shah A, Neupane S (2012). Evidence of HPV subtypes linked with cervical cancer in Nepal. WHO South East Asia J Public Heal.

[11] Shakya S, Syversen U, Åsvold BO, Bofin AM, Aune G, Nordbø SA (2017). Prevalence of human papillomavirus infection among women in rural Nepal. Acta Obstet Gynecol Scand.

[12] Johnson DC, Bhatta MP, Smith JS, Kempf MC, Broker TR, Vermund SH, et al. Assessment of high-risk human papillomavirus infections using clinician-Aand self-collected cervical sampling methods in rural women from far Western Nepal. PLoS One. 2014 [cited 2017 Jun 18];9. Available from: http://journals.plos.org/plosone/article?id=10.1371/journal.pone.0101255. 10.1371/journal.pone.0101255 PMC4076302 24978811

[13] Bhatta MP, Johnson DC, Lama M, Aryal S, Lhaki P, Shrestha S. High-risk human papillomavirus infection and abnormal cervical cytology among Nepali and Bhutanese refugee women living in eastern Nepal. BMC Infect Dis. 2017;17(73) Available from: http://bmcinfectdis.biomedcentral.com/articles/10.1186/s12879-017-2186-2 10.1186/s12879-017-2186-2 PMC5237500 28088173

[14] Carter JR, Ding Z, Rose BR (2011). HPV infection and cervical disease: a review. Aust. N. Z. J Obstet Gynaecol.

[15] Tantitamit T, Termrungruanglert W, Khemapech N, Havanond P (2017). A model approach for assessing the benefits of HPV testing against cytology in screening for cervical cancer precursors in Thailand. Asian Pacific J. Cancer Prev.

[16] Campos NG, Mvundura M, Jeronimo J, et al. Cost-effectiveness of HPV-based cervical cancer screening in the public health system in Nicaragua. BMJ Open. 2017;7:e015048. 10.1136/bmjopen-2016-015048. 10.1136/bmjopen-2016-015048 PMC5623348 28619772

[17] Central Bureau of Statistics (2012). National Population and Housing Census 2011 Central Bureau of Statistics.

[18] District Health Office. District Health Office Report. Jumla: District Health Office; 2011.

[19] Solomon D, Davey D, Kurman R, Moriarty A, O’Connor D, Prey M (2002). The 2001 Bethesda System: Terminology for Reporting Results of Cervical Cytology. JAMA.

[20] World_Health_Organization (2010). Human papillomavirus laboratory manual. first.

[21] Remmerbach TW, Brinckmann UG, Hemprich A, Chekol M, Kühndel K, Liebert UG (2004). PCR detection of human papillomavirus of the mucosa: comparison between MY09/11 and GP5+/6+ primer sets. J Clin Virol.

[22] Sanjosé S, Díaz M, Castellsagué X, Clifford G, Bruni L (2007). Worldwide prevalence and genotype distribution of cervical HPV in women with normal cytology. Lancet Infect.

[23] Coutlée F, Gravitt P, Kornegay J, Richardson H, Lapointe N, Franco E (2002). Use of PGMY primers in L1 consensus PCR improves detection of human papillomavirus DNA in genital samples use of PGMY primers in L1 consensus PCR improves detection of human papillomavirus DNA in genital samples. J Clin Microbiol.

[24] Wang R, Lei GX, GBA W, Schuuring E, Feng WW, Yu ZZ (2015). Nationwide prevalence of human papillomavirus infection and viral genotype distribution in 37 cities in China. BMC Infect Dis.

[25] Hamlin-Douglas LK, Coutlée F, Roger M, Franco EL, Brassard P (2008). Prevalence and age distribution of human papillomavirus infection in a population of Inuit women in Nunavik, Quebec. Cancer Epidemiol Biomark Prev.

[26] Kang L-N, Castle PE, Zhao F-H, Jeronimo J, Chen F, Bansil P, et al. A prospective study of age trends of high-risk human papillomavirus infection in rural China. BMC Infect Dis. 2014;14(96) Available from: http://bmcinfectdis.biomedcentral.com/articles/10.1186/1471-2334-14-96 10.1186/1471-2334-14-96 PMC3936871 24559293

[27] Kjaer SK, Chackerian B, Van Den BAJC, Kru S, Paull G, JMM W (2001). High-Risk Human Papillomavirus Is Sexually Transmitted : Evidence from a Follow-Up Study of Virgins Starting Sexual Activity (Intercourse). Cancer Epidemiol Biomark Prev.

[28] Camargo M, Soto-De Leon SC, Munoz M, Sanchez R, Peña-Herrera D, Pineda-Peña AC (2014). Human papillomavirus detection in women with and without human immunodeficiency virus infection in Colombia. BMC Cancer.

[29] Bhatla N, Lal N, Bao YP, Ng T, Qiao YL (2008). A meta-analysis of human papillomavirus type-distribution in women from South Asia: implications for vaccination. Vaccine.

[30] Clifford G, Smith J, Franceschi S, Plummer M, Munoz N. Human papillomavirus types in invasive cervical cancer worldwide: a meta-analysis. Br J Cancer. 2003:63–73. 10.1038/sj.bjc.6600688 PMC2376782 12556961

[31] Center for Disease Control and Prevention. HPV Vaccine Information for Clinicians; 2015. p. 1–5. Available from: https://www.cdc.gov/hpv/hcp/need-to-know.pdf. [cited 2017 Dec 23].

[32] Singh Y, Shah A, Singh M, Verma S, Shrestha BM, Vaidya P (2010). Human papillomavirus vaccination in Nepal: an initial experience in Nepal. Asian Pac J Cancer Prev.

